# A Generalized Linear Model for Estimating Spectrotemporal Receptive Fields from Responses to Natural Sounds

**DOI:** 10.1371/journal.pone.0016104

**Published:** 2011-01-11

**Authors:** Ana Calabrese, Joseph W. Schumacher, David M. Schneider, Liam Paninski, Sarah M. N. Woolley

**Affiliations:** 1 Doctoral Program in Neurobiology and Behavior, Columbia University, New York, New York, United States of America; 2 Center for Theoretical Neuroscience, Columbia University, New York, New York, United States of America; 3 Department of Statistics, Columbia University, New York, New York, United States of America; 4 Department of Psychology, Columbia University, New York, New York, United States of America; University of Auckland, New Zealand

## Abstract

In the auditory system, the stimulus-response properties of single neurons are often described in terms of the spectrotemporal receptive field (STRF), a linear kernel relating the spectrogram of the sound stimulus to the instantaneous firing rate of the neuron. Several algorithms have been used to estimate STRFs from responses to natural stimuli; these algorithms differ in their functional models, cost functions, and regularization methods. Here, we characterize the stimulus-response function of auditory neurons using a generalized linear model (GLM). In this model, each cell's input is described by: 1) a stimulus filter (STRF); and 2) a post-spike filter, which captures dependencies on the neuron's spiking history. The output of the model is given by a series of spike trains rather than instantaneous firing rate, allowing the prediction of spike train responses to novel stimuli. We fit the model by maximum penalized likelihood to the spiking activity of zebra finch auditory midbrain neurons in response to conspecific vocalizations (songs) and modulation limited (ml) noise. We compare this model to normalized reverse correlation (NRC), the traditional method for STRF estimation, in terms of predictive power and the basic tuning properties of the estimated STRFs. We find that a GLM with a sparse prior predicts novel responses to both stimulus classes significantly better than NRC. Importantly, we find that STRFs from the two models derived from the same responses can differ substantially and that GLM STRFs are more consistent between stimulus classes than NRC STRFs. These results suggest that a GLM with a sparse prior provides a more accurate characterization of spectrotemporal tuning than does the NRC method when responses to complex sounds are studied in these neurons.

## Introduction

Characterizing neural responses to natural stimuli remains one of the ultimate goals of sensory neuroscience. However, considerable technical difficulties exist for correctly estimating neural receptive fields (RFs) from natural stimuli. Two major difficulties are the interactions between higher-order statistics of the stimuli and inherent nonlinearities of neural responses [1], [2] and the challenge of estimating receptive fields in high dimensional spaces with limited data [3], [4].

Neural responses are commonly characterized by a linear-nonlinear (LN) model [5]–[7], in which the output of a linear filter or receptive field (RF) applied to the stimulus is then transformed by a static nonlinearity to determine the instantaneous firing rate of the neuron. Reverse correlation (RC), the most widely used estimation method, computes the RF of a neuron by multiplying the spike-triggered average (STA) of the stimulus by the inverse of the stimulus covariance matrix. It is well understood that, for an LN neuron, RC is guaranteed to produce an unbiased estimate of a neuron's true underlying filter only if the distribution of the stimuli used for estimation is elliptically symmetric [6]. Deviations from either the LN framework (e.g., the existence of more than one linear filter (multiple-filter LN), or extra terms that take into account spiking history) or the elliptical symmetry condition (e.g., naturalistic stimuli which contain higher order correlations) can introduce biases in the estimate of the RF.

The highly correlated structure of natural stimuli presents additional numerical problems for RF estimation. Because natural stimuli contain strong autocorrelations, the majority of the power in the stimulus tends to be concentrated in a small number of dimensions. Multiplication by the inverse of the stimulus covariance matrix causes noise in the resulting RF to be strongly amplified along the stimulus dimensions with low variance. Thus, the RC fitting algorithms are adjusted (i.e. regularized) with a “regularizing” operation or term to prevent overfitting to noise [3], [8]–[10].

In the auditory system, the stimulus-response properties of single neurons are often described in terms of the spectrotemporal receptive field (STRF), a linear kernel relating the spectrogram of the sound stimulus to the instantaneous firing rate of the neuron. Traditionally, STRFs have been estimated using normalized-reverse correlation (NRC), a method that uses an approximation to the stimulus covariance matrix to obtain regularized estimates. Regularization introduces a prior that imposes constraints in the STRF estimate and, under noisy conditions, the specific regularization used by the model can introduce biases in the estimates [4]. Methods other than NRC have recently been proposed to characterize the tuning properties of auditory neurons from responses to natural stimuli, each of which reduces the impact of stimulus-correlation biases on the estimated STRFs [4], [8], [11]. These algorithms differ in their functional models, cost functions, and regularization methods. Here, we propose an approach for characterizing the stimulus-response function of auditory neurons based on a generalized linear model (GLM). This method is advantageous because it requires relatively light computational resources and provides easily interpretable results [12]–[14]. For example, it has been successfully used to accurately predict spiking responses of single retinal ganglion cells [15] as well as detailed spatiotemporal correlations in the responses of a complete population of macaque retinal ganglion cells [16]. As opposed to most STRF estimation methods, our method takes into account spiking history. Further, the output of the model is a series of spike trains rather than average time-varying firing rate, allowing comparison of the actual and predicted spike train responses.

In this study, we compare a GLM with a sparse prior and NRC in terms of their ability to predict responses to novel stimuli and the tuning properties of the STRFs they produce. We fit both models to responses of single auditory neurons in the midbrain of zebra finches probed with two types of stimuli: zebra finch songs and modulation-limited noise. We find that the GLM predicts responses to both stimulus classes significantly better than NRC, and that GLM and NRC STRFs derived from the same data can differ profoundly. Finally, the GLM method reduces differences in tuning between stimulus classes.

## Materials and Methods

### Ethics Statement

Birds were cared for in accordance with the US National Institutes of Health Guide for the Care and Use of Laboratory Animals. All procedures were approved by the Columbia University Institutional Animal Care and Use Committee.

### 2.1 Stimuli and average response properties

Two sound ensembles were used: a conspecific song ensemble and a modulation-limited (ml) noise ensemble. The conspecific song ensemble consisted of 20 songs (∼2 sec in duration each) from different adult male zebra finches. Each song was band-pass frequency filtered between 250 Hz and 8 kHz. The ml noise ensemble consisted of 10 samples of 2 sec of ml noise. Ml noise is a behaviorally meaningless sound similar to white noise that was designed to match song in frequency range, maximum spectral and temporal modulations and power [17]. Stimuli were played through a flat frequency response speaker positioned at 20 cm in front of the bird, at a mean intensity of 72 dB SPL. This stimulus intensity is comparable to behavioral sound levels during singing and song perception [18]–[20] and is above the pure tone threshold for auditory neurons in MLd [21]. Ten spike train response trials were obtained for each of the 20 songs and 10 noise samples. Trials for different stimuli were interleaved in random order. The inter-trial interval was determined at random from a uniform distribution between 1.2 and 1.6 seconds.

### 2.2 Electrophysiology

Electrophysiological recordings were made from single neurons in the auditory midbrain, mesencephalicus lateralis dorsalis (MLd), of adult male zebra finches as described in [22]. Briefly, an initial preparatory surgery was performed 48 hrs before the first neural recording session. Birds were deeply anesthetized with 0.03 ml Equithesin and placed in a custom stereotaxic holder. For recordings made from anesthetized birds, only the first layer of skull was removed during the initial surgery. For recordings made from awake birds, full craniotomies were made. A grounding wire was cemented in place with its end just beneath the skull, approximately 5 to 10 mm lateral to the junction of the midsagittal sinus. A head post was cemented to the skull of the animal and points were marked for electrode penetrations. Anesthetized recording sessions were preceded by administering three doses of 0.03 ml of 20% urethane over a period of one hour. Recordings were made using glass pipettes containing 1M NaCl, with impedances ranging from 5 to 20 MOhms. The duration of the recording sessions ranged from 4 to 15 hours. Awake recording sessions were no longer than 6 hours. For a single animal, awake recordings were performed over a period of approximately two weeks and anesthetized recordings were performed in a single session. After final recording sessions, the birds were euthanized and brains were preserved for histological reconstruction of electrode locations.

We recorded from 169 well-isolated MLd neurons (97 in anesthetized birds and 72 in awake birds). Neurons recorded from awake and anesthetized birds produced robust responses to songs and ml noise. On average, midbrain neurons recorded from awake birds showed higher spontaneous and stimulus-driven firing rates, when compared to neurons recorded from anesthetized birds (mean stimulus-driven firing rates were 22 Hz for the awake preparation and 11 Hz for the anesthetized preparation). In accordance with previous studies [17], [23] we did not find significant differences in mean (spontaneous or driven) firing rate in responses to song and ml noise in awake or anesthetized birds, at the single cell level. The responses of nearly all neurons were stimulus-locked and reliable over multiple presentations of the same stimulus (trials).

### 2.3 Data preprocessing

The same preprocessing was applied to the data before fitting both NRC and GLM. Spectrograms were generated from the stimulus sound pressure waveforms using a bank of band-pass filters with center frequencies ranging from 250 to 8000 Hz, which covers the audible frequency range for zebra finches [24]. The center frequencies were spaced linearly and had a bandwidth of 125 Hz. It has been shown that the predictive abilities of STRFs can be improved by applying a compressive nonlinearity to the stimulus spectrogram [25]. We therefore applied a logarithm to the stimulus spectrogram prior to fitting the models, which mimics peripheral auditory processing.

For the NRC method, both stimulus spectrograms and spike trains were binned at 1 ms resolution (the temporal resolution required by STRFPak, the publicly available Matlab toolbox for STRF estimation we used in this study; see Section 2.5). For the GLM method, both signals were further down sampled by a factor of 3. Using time bins larger than 1 ms is common in the GLM setting [26]. Expanding the bin size can avoid nonconvergence problems related to the refractory periods of neurons [27], and effectively reduces the computational load. In order to ensure that the different bin sizes in the estimation of NRC and GLM STRFs did not introduce a bias in predictive power or STRF shape, we re-computed the STRFs of a subset of our population of cells (10 cells) using the GLM with 1 ms time bins. We found no significant differences in STRF shape or predictive power of GLM STRFs computed at 1 ms or 3 ms resolution (the average same-class prediction correlation for a novel song was 0.507 and 0.51, respectively).

### 2.4 Generalized linear model for spike trains

**Figure 1:**
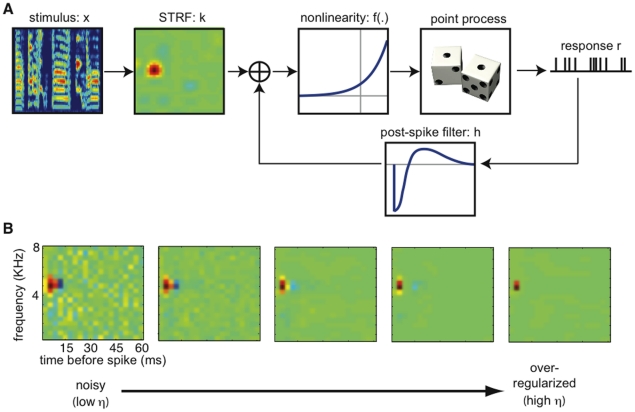
Methods. (**A**) Generalized linear model (GLM) schematic. Each neuron has a stimulus filter or STRF (*k*), and a post-spike filter (*h*) that captures dependencies on the cell's own spiking history. Summed filter output passes through a static nonlinearity *f* to produce the instantaneous spike rate. (**B**) Illustration of the effect of a sparse prior on the STRF estimate. Panels from left to right show STRFs estimated by maximum penalized likelihood for increasing values of the penalization parameter η. Low values of η lead to noisy estimates. For very high values of η, very few parameters are nonzero. The optimal value of η is determined by cross-validation STRFs have been plotted in their raw, low resolution state.

We describe the encoding of a stimulus spectrogram (a transformation of the sound pressure waveform into a time-varying function of energy in each frequency band), 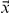, in the spike trains of an auditory neuron with a generalized linear model (GLM, Figure 1A), a generalization of the well known linear-nonlinear-Poisson (LNP) cascade model [12]. In this model, a cell's response is described by: 1) a stimulus filter, or STRF (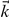); 2) a post-spike filter (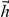), which captures dependencies on the cell's spiking history (e.g. refractoriness); and 3) a constant offset *b* which sets the baseline firing rate of the model. For each neuron, a static nonlinear function is then applied to the summed filter responses to obtain an instantaneous spike rate [13], [14], [28]. Although these types of models are strictly phenomenological, their components can be broadly compared to biophysical mechanisms. The stimulus filter approximates the spectrotemporal integration of the sound stimulus in an auditory neuron. The post-spike filter mimics voltage-activated currents following a spike. And the output nonlinearity implements a soft threshold converting membrane potential to instantaneous spike probability.

#### 2.4.1 Model fitting/parameter estimation

We fit the model to extracellular single unit recordings from 169 auditory midbrain neurons. To calculate the model parameters, stimuli (log spectrograms) were computed and responses (spike trains) were binned at a 3 ms resolution. The time bin was small enough that more than one spike was almost never observed in any bin. The spectral domain of the stimulus was divided into 20 equally spaced frequency bins, which spanned frequencies between 250 and 8000 Hz. The parameters for each cell consisted of a stimulus filter or STRF 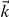, a constant offset *b*, and a post-spike filter 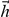. The STRF was a 400-dimensional vector (20 spectral ×20 time bins, including frequencies between 250 and 8000 Hz and latencies between 0 and 60 ms, respectively), the post-spike filter was a 5-dimensional vector (5 time bins spanning the 15 ms following each spike) and the offset consisted of a scalar value, for a total of 406 unknown parameters. All the model parameters (*b*, 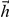, and 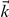) were fitted simultaneously by maximum penalized likelihood. Increasing the binning resolution would change the number of fit parameters and could, in theory, improve the performance of the model. However, the resolution used in this study is sufficient for demonstrating the performance of GLM compared to NRC under these experimental conditions.

The conditional spike rate in the model is given by 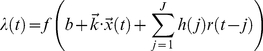
(1)where 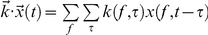 is the convolution between the stimulus at time *t* and the STRF, and *r(t-j)* is the cell's spike train history (*J = 5*). In the case in which the nonlinearity *f* is an exponential function, the offset *b* corresponds to the log-probability of spontaneous firing of the cell. The log-likelihood of the observed spike data given the model parameters (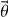 =  {*b*, 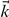, 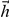}), and the observed stimulus 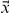 is given by the point process log-likelihood [29]

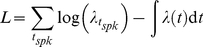
(2)where *t_spk_* denotes the set of spike times and the integral is taken over the length of the experiment (all trials of the particular stimulus class used to fit the model).

#### 2.4.2 Static nonlinearity

Numerical optimization of Eq. 2 is simplified by making two assumptions about the nonlinear rectification function f (.): 1) f (*u*) is a convex function of its scalar argument *u*; and 2) log f (*u*) is concave in *u*. With these assumptions, the log-likelihood in Eq. 2 is guaranteed to be a concave function of the parameters 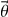 [30]. This ensures that the likelihood has no local maxima, and therefore the maximum likelihood parameters 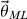 may be found by numerical ascent techniques. Several functions *f(.)* satisfy these two constraints, including the standard linear rectifier and the exponential function.

**Figure 2:**
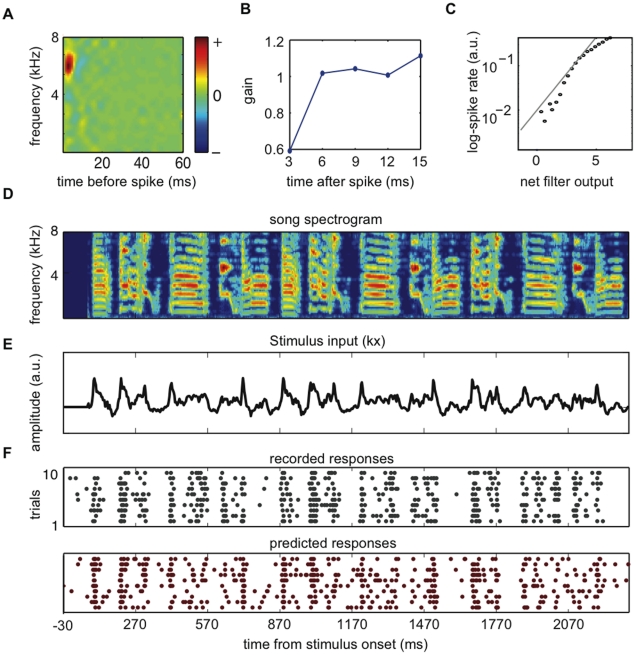
A GLM as a neural encoding model. (**A**)–(**B**) Estimated parameters for an example auditory midbrain neuron. (**A**) STRF. (**B**) Exponentiated post-spike filter, which may be interpreted as a spike-induced gain adjustment of the neuron's firing rate. It produces a brief refractory period and gradual recovery (with a slight overshoot). (**C**) Estimate of the nonlinearity transforming linear input to instantaneous spike rate (black points), for the same example neuron (Chichilnisky 2001). The nonlinearity represents the probability of observing a spike for each value of net linear input (b+k*x+h*r). An exponential function (grey line), the assumed nonlinearity for the model, provides a reasonable approximation to this function. (**D**) Spectrogram (*x*) of one example song used in the experiments. (**E**) Stimulus filtered by STRF, k*x. (**F**) Recorded (gray) and predicted (red) raster plots in response to the validation stimulus shown in (**D**).

For each cell, our model converts linear input into an instantaneous spike rate by means of an exponential nonlinearity (see Figure 1A). To assess the adequacy of this assumption, we compared an exponential function with a direct reconstruction estimate of the nonlinearity, computed using the raw distribution of filter outputs and the observed spike responses [5] (see Figure 2C for an example). These reconstructions look exponential for some cells in our dataset and sub-exponential for others. To assess the performance of the exponential nonlinearity against another nonlinearity, we also performed a complete re-fitting of the model parameters using output nonlinearities given by a function of the form 
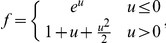



which grows quadratically for *u>0* and decays like *e^u^* for negative values of *u*. This model exhibited slightly better predictive power for ml noise (p<0.05, two-sample Kolmogorov-Smirnov test) but not for songs across the population of 169 cells, and did not result in a noticeable change in the fitted STRFs.

The weak dependence of the parameter estimates on the specific form of the nonlinearities tested here (in addition, we also fitted a linear model with a sparse prior which resulted in nearly identical STRFs, see Section 3.6) led us to ask whether we could improve the performance of the model by fitting a flexible nonlinearity for each cell once the parameters (b, 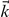, and 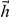) were already known (in general, the estimates will depend on the specific form of the objective function used for optimization and a re-estimation step is necessary after the nonlinear function *f* is fitted). We parameterized the output nonlinearity as a cubic spline, and used this model instead of the exponential nonlinearity to predict novel responses and compared those to predicted responses that were generated using the exponential nonlinearity. This addition conferred only a slight improvement in cross-validation performance (see Section 3.6). Therefore, for simplicity, we restricted all further analyses in this study to a GLM with an exponential nonlinearity.

#### 2.4.3 Regularized sparse solutions

Maximum likelihood estimates can be extremely noisy when fitting high-dimensional models. This overfitting phenomenon has been shown in the linear regression case [3] (see section 2.5), where the noisiness of the estimate of the filter
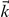
is roughly proportional to the dimensionality of
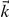
divided by the total number of observed samples [6]. The same type of effect occurs in the GLM context. Thus, in order to obtain accurate fits, we added to the log-likelihood in Eq. 2 an additional term, *Q*(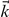), that acts as a “penalty function.” 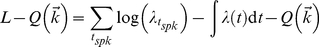
(3)


Here *Q*(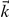) encodes our a priori beliefs about the true underlying 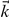. Whenever the penalizer −*Q*(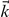) is a concave function of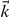, the penalized likelihood in Eq. 3 is also a concave function of 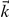, and ascent-based maximization may proceed as before, with no local maxima [30]. Thus, the penalty term *Q* can be any function within the class of convex functions.

Here we used a sparse prior on the STRF (i.e., many of the elements of 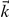
are zero and only a small subset of the elements of 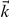 is active) to regularize the model. This is equivalent to assuming that the neuron's firing is sensitive only to a small number of stimulus features [4]. A common way to impose sparseness is based on the L1 norm of 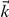 [31], [32],
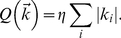
(4)


This function is convex, but the term on the right in Eq. 4 is non-differentiable and the resulting optimization problem can be challenging. An alternative approach is to use a smooth differentiable approximation to the L1-regularizer that would allow the application of standard Newton methods to solve the resulting unconstrained optimization problem. Within this context, we use the interior point method proposed by [33] to solve the optimization problem. This method relaxes the non-differentiability of the L1-norm by a sequence of smooth approximation functions. Solving this optimization problem requires the selection of an additional hyperparameter, η, that controls the amount of penalization: for large η we penalize strongly and for η = 0 we recover the maximum likelihood unregularized solution (Figure 1B). Here, we select this hyperparameter by cross-validation, varying η until a maximum in prediction accuracy is reached.

### 2.5 STRF estimation by normalized reverse correlation

For comparative purposes, we estimated STRFs from the same data using normalized reverse correlation (NRC), a variant of the classical linear regression that has been used to estimate STRFs from natural stimuli in the auditory and visual systems [3], [34], [35]. NRC fits a linear STRF that minimizes the mean-squared error between predicted and observed neuronal response:
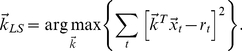
(5)


A detailed description of the algorithm is described in [3]. Here we provide a brief description of NRC for comparison to the GLM method.

The best-fit least-squares filter 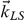 is given by
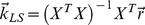
(6)where the stimulus matrix *X* is defined as *X_t_ = x_t_^T^* and *r = (r(1) r(2)* … *r(t))^T^*. The term 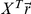 corresponds to the spike-triggered average - the conditional mean 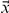 given a spike - and the matrix *X^T^X/D = C_XX_* corresponds to the covariance matrix of the stimulus. Here, the superscript *T* indicates a transpose operation and *D* is the duration of the experiment.

This estimator gives an unbiased estimate of the filter 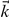 for any stimulus statistics if the underlying system is linear [36] or if the stimulus is elliptically symmetric (i.e. contains only up to second order correlations) if the underlying system is nonlinear. However, in practice, for the case of high dimensional *X* with strong autocorrelations, estimating 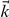 with Eq. 6 can amplify noise excessively [3]. To minimize these effects, NRC uses a pseudo inverse to approximate the inverse of the stimulus autocorrelation matrix. This approximation is based on setting dimensions in the stimulus that have little power (below some noise threshold) to zero. To compute the pseudo inverse, a singular value decomposition is applied to the autocorrelation matrix,
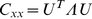
(7)


The columns of U contain the unit-norm eigenvectors of *C_XX_*, which correspond to the discrete Fourier transform (DFT) vectors. The diagonal matrix *Λ =  diag(λ_1_, λ2,* … , *λ_N_)* contains the corresponding eigenvalues ordered from largest to smallest, which correspond to the power spectrum of the stimulus as a function of temporal frequency. A tolerance value, τ, specifies the fraction of stimulus variance and the number of stimulus dimensions, *m*, to preserve in the pseudoinverse *C^−1^_app_:*

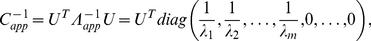
(8)which results in penalization of high frequencies.

The final NRC estimate of the STRF is then, 
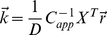
(9)


Implementing NRC requires the selection of a single hyperparameter, the tolerance value, τ. To choose τ, the method uses a cross-validation procedure. In this study, we use the Matlab toolbox developed by the Theunissen and Gallant laboratories at University of California, Berkeley (http://strfpak.berkeley.edu) to estimate NRC STRFs.

### 2.6 Prediction accuracy

For both the GLM and NRC models, response prediction was validated with song and noise data that were not used for fitting the model. From the entire set of 20 song and 10 ml noise stimuli, 19 songs or 9 ml noise samples were used to estimate the models' parameters (estimation data set). The models were then used to predict the average response to the remaining song or ml noise sample not included in the estimation set. This procedure was repeated 20 times in the case of songs and 10 times in the case of ml noise, excluding a different validation stimulus on each repeat. This analysis ensures that no individual song (ml noise) sampled can bias the STRF calculations or the quantification of prediction accuracy because the responses to each song (ml noise) have been removed from the STRF calculation once and the responses to each song (ml noise) have been used as the testing set once. The 20 (10) accuracies of these predictions were then averaged to produce a single value representing the prediction accuracy of a given model (NRC or GLM) in response to songs or ml noise.

Prediction accuracy was determined by measuring Pearson's correlation coefficient between the predicted and observed peristimulus time histogram (PSTH). For both NRC and GLM, PSTHs were computed with a 3 ms time bin and were smoothed with a 5 ms Hanning window. The width of the smoothing window was chosen to match the integration time of auditory midbrain neurons [37].

### 2.7 Tuning properties derived from STRFs

In addition to comparing the GLM and NRC methods in terms of their predictive power where the complete STRFs (both their inhibitory and excitatory portions) were used for response estimation, we parametrically compared STRFs from both methods using three measures of tuning properties commonly used to characterize auditory neurons [23], [38]; 1) best excitatory (inhibitory) frequency, eBF (iBF), the spectral frequency that evokes the strongest (weakest) neural response; 2) excitatory (inhibitory) spectral bandwidth, eBW (iBW), the range of frequencies that are associated with an increase (decrease) from mean firing rate; and 3) excitatory (inhibitory) temporal bandwidth, etBW (itBW), the time over which relevant frequencies lead to an increase (decrease) from mean firing rate. eBF, iBF, eBW, iBW, etBW, and etBW were computed from the STRFs using standard methods [39]. Briefly, the eBF (iBF) was measured by setting negative (positive) STRF values to zero and averaging along the time axis. The resulting spectral tuning curve was convolved with a 5-point symmetric Hanning window, and the eBF (iBF) was taken to be the position of the peak (valley) of the smoothed curve. The eBW (iBW) was measured from the smoothed curve as the width (Hz) at half-height (half-depth) around the eBF (iBF). The etBW (itBW) was measured by setting all negative (positive) STRF values to zero and averaging along the spectral axis. The resulting temporal tuning curve was convolved with a 5-point symmetric Hanning window, and the etBW (itBW) was measured from the smoothed curve as the width (ms) at half-height (half-depth) around the peak.

## Results

### 3.1 Responses of single auditory midbrain neurons are well modeled using a GL model

We modeled the functional relationship between sound stimuli and neuronal responses with a generalized linear model (GLM) for each neuron (see Figure 1A). Figure 2A shows an example GLM STRF estimated from responses to song and Figure 2B shows the corresponding exponentiated post-spike filter representing the influence of spiking history on spiking probability for the same neuron. For most of the cells in our sample, the shape of the post-spike filter corresponds to a brief period of refractoriness (5.3+/−0.2 ms, mean +/− SE) and gradual recovery (see Figure 2B for an example).

Figure 2C shows the static nonlinearity estimated for this neuron [5] (black dots), together with the exponential nonlinearity (gray line) employed by the model. Although the exponential function used by the model does not provide an excellent fit to the underlying nonlinearity for this neuron (a subexponential nonlinearity performs slightly better; see Methods), the model does predict responses to a novel stimulus with good accuracy (see below).

In order to test how well the GLM method predicted song responses in individual trials, we used it to predict the responses to a validation song (Figure 2D–F) that was included in the recording experiment but was not included in the estimation of the model parameters. Recorded and predicted spiking responses to the validation stimulus are shown in Figure 2F. For this neuron, the model predicts the spiking responses to the validation song reasonably well; the mean cross correlation between actual and predicted response PSTHs was 0.69.

### 3.2 A GLM outperforms normalized reverse correlation (NRC) when predicting responses of single auditory neurons to songs and noise

We next compared the GLM to the more traditional STRF estimation method, NRC, in the ability to predict single neuron responses to zebra finch songs and ml noise.

**Figure 3:**
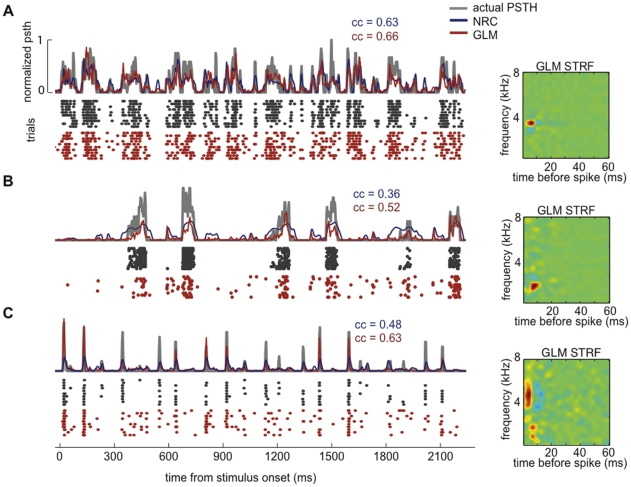
The GLM outperforms NRC when predicting responses to songs. (**A**)–(**C**) Examples of NRC and GLM response prediction (PSTHs and spike trains) and corresponding GLM STRFs for three auditory midbrain neurons. Recorded and predicted responses correspond to the song shown in Figure 2D. Spike trains and PSTHs were computed with a 3 ms time bin and PSTHs were smoothed with a 5 ms Hanning window prior to computing correlation coefficients. (STRFs have been up sampled by a factor of 3 for visualization). The GL model performs consistently better than NRC when used to predict average responses to a validation stimulus. In addition, the GLM spike-train predictions capture the overall structure of the actual spike trains.

Figure 3 shows NRC and GLM response predictions for three neurons in response to the song in Figure 2D. Although the predicted traces for both models (blue for NRC and red for GLM) account for broad variations in the actual PSTHs, neither of them captures their precise shape. One common failure of the models to predict responses is best demonstrated in Figure 3B and C. These two neurons show highly reliable responses to the song and, although the models predict the timing of the responses, in several cases they underestimate their amplitudes. This effect is more pronounced for NRC than for the GLM. Changing the nonlinear link function of the model (from an exponential to another type of nonlinearity, see Eq. 1 and Section 2.4.2) could, in principle, help to increase the amount of variance in the response described by the model. However, within the groups of nonlinearities we tried on our data (see Methods), we observed only slight or no improvements in prediction accuracy. We later discuss (see Discussion) several extensions to the GLM that could improve the predictive power of the model. Finally, Figure 3 shows spike-train predictions for the GLM method in response to the same song. The predicted spike trains capture the overall structure of the recorded spiking activity.

We then compared the prediction accuracy of the GLM and NRC methods across the entire set of 169 auditory midbrain neurons. Since we did not find noticeable differences in predictive power between awake and anesthetized recordings, we report the prediction accuracy for the two data sets combined.

**Figure 4:**
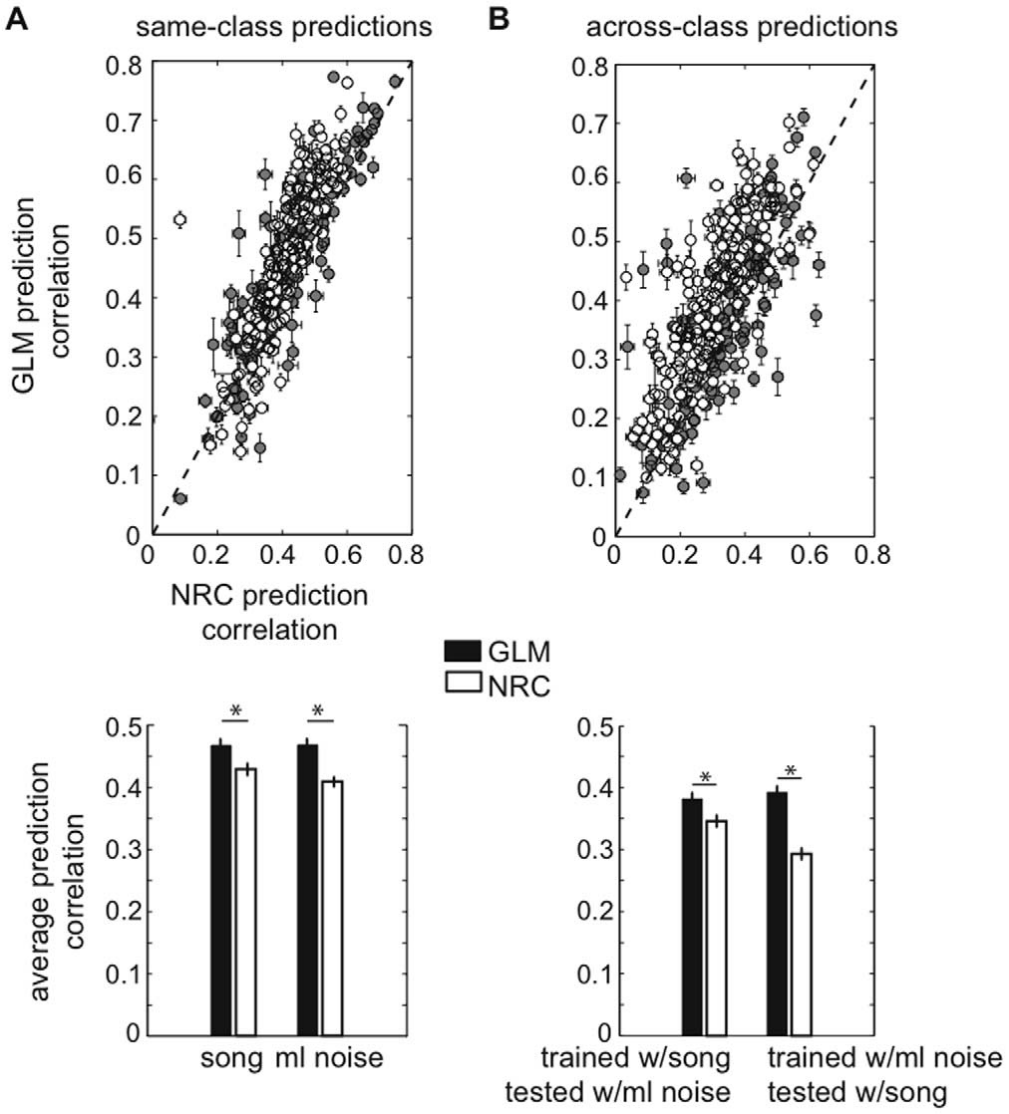
The GLM has higher predictive power than does NRC within- and across stimulus classes. Both methods were evaluated by their ability to predict responses to a validation song and ml noise data set that was not used for parameters' estimation. (**A**) Same-class predictions: NRC and GLM were used to predict responses to a novel song or ml noise stimulus when only songs or ml noise were used to train the model. (**B**) Across-class predictions: NRC and GLM were used to predict responses to a novel song or ml noise stimulus when the other stimulus ensemble was used to train the model. Each point plots the correlation coefficient between the observed and predicted average response (PSTH), for NRC (horizontal axis) and GLM (vertical axis) for a single neuron. White dots indicate responses to ml noise and gray dots indicate responses to song. We found that on average, the GLM predicts responses significantly better than NRC (*p<0.001, two-sample KS test) both in the same-class and across-class cases. Error bars represent SEs.

We first compared the ability of both models to predict responses to a novel stimulus taken from the same stimulus class used in the estimation set (we refer to this case as “same-class predictions”, Figure 4A). We found that the performance of both models varies widely across our population of cells; on this moderately small timescale (predicted and actual responses were computed using 3 ms time bins and were smoothed with a 5 ms Hanning window; see Methods), the prediction correlation was as high as 0.77 for some neurons and below 0.1 for others. For low firing rate neurons, we found a relatively moderate correlation (0.36) between the number of spikes in the estimation set and the prediction performance of the models. We found that the prediction performance becomes independent of the number of spikes in the estimation set for N ∼2000, which corresponds to firing rates of ∼10 Hz. Since the goal of this study is to test the GLM method under different conditions and compare its performance to NRC, we included all the data in our sample in the analysis regardless of prediction accuracy.

The average same-class prediction correlation for the GLM for novel song and ml noise stimuli is *r_s_ = 0.47±0.01* and *r_n_ = 0.46±0.01*, respectively (mean ± SE). These values are significantly greater than the average for NRC, *r_s_ = 0.42±0.01* and *r_n_ = 0.40±0.008* (*p<0.001*, two-sample Kolmogorov-Smirnov (KS) test).

To evaluate how well the GLM and NRC methods estimated from responses to one of the stimulus classes generalized to a second stimulus domain, we compared how well these models predicted responses to the other stimulus class (“across-class predictions”). In this way, we used the models that were estimated using song data to predict responses to ml noise and vice-versa (Figure 4B). As in the case of same-class predictions, the GLM predicts responses to the opposite class (*r_s_ = 0.38±0.01* and *r_n_ = 0.4±0.01*) significantly better than NRC (*r_s_ = 0.34±0.01* and *r_n_ = 0.29±0.01*, *p<0.001*, two-sample KS test).

The absolute prediction accuracy for both models in the across-class case is significantly lower than in the same-class case. For the GLM, the mean prediction correlation is 15% lower in the across-class case than in the same-class case, both for noise and song predictions. For NRC, the mean prediction correlation is 15% lower in the across-class case than in the same-class case for song predictions, and 28% lower for noise predictions. This decrease in performance suggests that neither model generalizes completely to other stimulus classes. Because of nonlinear response properties, STRFs estimated using one stimulus class tend to predict responses to other stimulus classes with worse accuracy [1], [23]. However, the better performance of the GLM suggests that it provides a more general characterization of spectrotemporal tuning across different stimulus conditions.

### 3.3 GLM STRFs are more stable to changes in the stimulus statistics than are NRC STRFs

**Figure 5:**
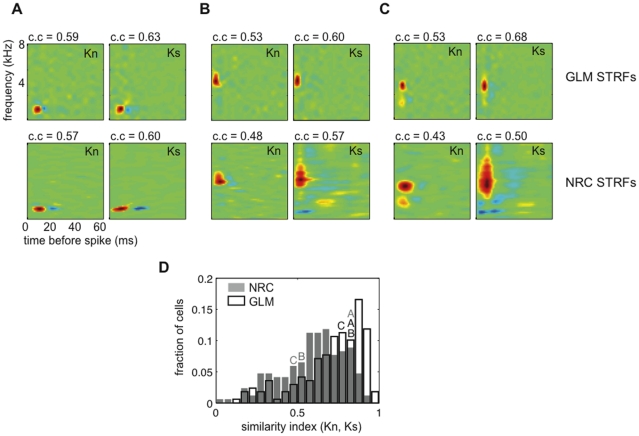
GLM STRFs are more similar across stimulus classes than are NRC STRFs. (**A**)–(**C**) Top row: GLM STRFs estimated from recorded responses to ml noise (Kn) and song (Ks) for three example midbrain auditory neurons. Bottom row: ml noise (Kn) and song (Ks) NRC STRFs for the same three cells. (**A**) An example cell for which Kn and Ks do not differ, both for the GLM and NRC. (**B**)–(**C**) Two example cells for which Kn and Ks differ significantly for NRC, but differ much less for the GLM. (**D**) Distributions of similarity indices (SIs) computed between Kn and Ks for NRC (grey) and GLM (white) for the population of 169 cells. One indicates an exact match between STRFs and 0 indicates no correlation. The GLM produces STRFs that are more similar across stimulus classes as seen by the shift to the right of the SI distribution (median of the SI distribution for the GLM was 0.76 as opposed to 0.61 for NRC). GLM and NRC distributions differ significantly (p<0.001, two-sample KS test). Letters indicate example STRFs.

The fact that the GLM produces better response predictions across stimulus classes than does NRC (see Figure 4B), suggests that it generalizes better to changes in the statistics of the stimulus used to estimate the model. In agreement with this, we found that GLM STRFs were more similar to each other between stimulus classes than NRC STRFs for the entire population of 169 cells. Figure 5A–C shows GLM (top panel) and NRC (bottom panel) STRFs derived from responses to song (Ks) or ml noise (Kn) for three example neurons. In agreement with previous observations [1], [23], [35], we found that, for some neurons, NRC STRFs estimated from different stimulus classes show significant differences (see, for example Figure 5B and 5C). Figure 5A shows an example neuron for which Kn and Ks do not differ, and Figure 5B–C shows example cells for which NRC STRFs estimated from recorded responses to ml noise and song differ significantly. In contrast, GLM song and noise STRFs appear significantly more similar for all three cells.

To quantify the differences between song and ml noise STRFs (Kn and Ks) derived using a GLM or NRC, we measured a similarity index (SI, pixel by pixel correlation). A similarity index of 1 indicates a perfect match, and an index of 0 indicates no correlation between STRFs. Figure 5D shows the distributions of SIs between Kn and Ks for the GLM (white) and NRC (grey) for the set of 169 neurons. These distributions differ significantly (*p<0.001* KS test). The shift to the right in the SI distribution for the GLM shows that GLM STRFs are more similar across stimulus classes than are NRC STRFs at the population level (median of the SI distribution 0.76 for the GLM and 0.61 for NRC).

In addition, we found that differences between NRC and GLM STRFs derived from responses to ml noise (compare GLM Kn vs. NRC Kn in Figure 5A–C) were smaller than differences between NRC and GLM STRFs derived from responses to songs (compare GLM Ks vs. NRC Ks in Figure 5A–C), as would be predicted theoretically. When stimuli that contain only second-order correlations are used to derive the STRF, NRC and GLM should give the same answer in the limit of infinite data [5], [6], [11]. Non-Gaussian effects in the ml noise stimulus ensemble are smaller than in the song ensemble, which explains the smaller differences between GLM and NRC STRFs for this stimulus class.

### 3.4 Tuning properties of GLM and NRC STRFs

**Figure 6:**
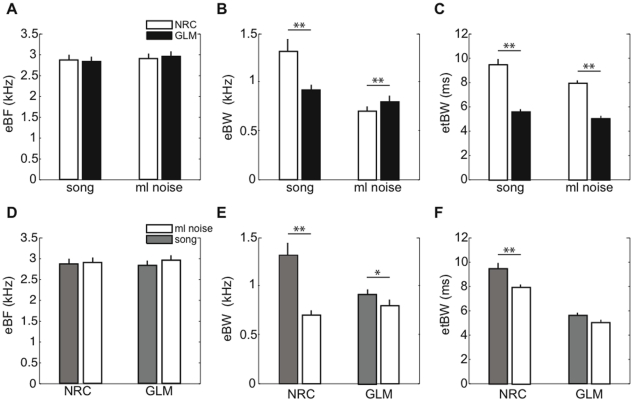
Excitatory tuning differences between GLM and NRC STRFs. (**A**)–(**C**) Comparison of excitatory tuning properties of GLM and NRC STRFs. (**A**) Best frequency of the excitatory region (eBF), (**B**) excitatory spectral bandwidth (eBW), and (**C**) excitatory temporal bandwidth (etBW). We found no significant differences in eBF between NRC and GLM STRFs derived from neural responses to ml noise, or those derived from responses to song (*p>0.9*, two-sample KS test). However, we found that differences in eBW and etBW determined by the estimation algorithms were highly significant (**p<10^-3^, two-sample KS test). (**D**)–(**E**) Comparison of excitatory tuning properties of song and noise STRFs. (**D**) eBF, (**E**) eBW and (**F**) etBW. We found no significant difference in eBF between song and noise STRFs in ether of the models (*p>0.9*). Differences in eBW between song and noise STRFs were considerably larger for NRC than for GLM (**p<10^−3^ and *p<0.05). Finally, we found significant differences between song and noise STRFs in terms of etBW for NRC but not for the GLM (*p<0.05 and *p>0.1*, respectively). Error bars represent SEs.

**Figure 7:**
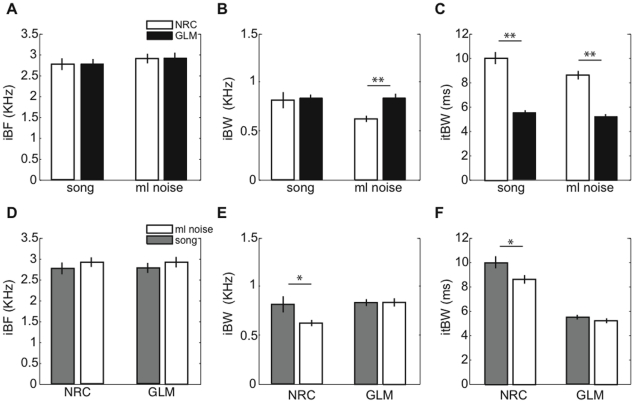
Inhibitory tuning differences between GLM and NRC STRFs. (**A**)–(**C**) Comparison of inhibitory tuning properties of GLM and NRC STRFs. (**A**) Best frequency of the excitatory region (iBF), (**B**) excitatory spectral bandwidth (iBW), and (**C**) excitatory temporal bandwidth (itBW). We found no significant differences in iBF between NRC and GLM STRFs derived from neural responses to ml noise, or those derived from responses to song (*p>0.8*, two-sample KS test). However, we found that differences in iBW determined by the estimation algorithms were significant for noise but not for songs (**p<10^−3^, two-sample KS test), and differences in itBW were highly significant both for songs and noise (**p<10^−3^, two-sample KS test). (**D**)–(**E**) Comparison of inhibitory tuning properties of song and noise STRFs. (**D**) iBF, (**E**) iBW and (**F**) itBW. We found no significant difference in iBF between song and noise STRFs in ether of the models (*p>0.1*). Differences in iBW between song and noise STRFs were significant for NRC (*p<0.05) but not for the GLM (*p>0.8*). Finally, we found significant differences between song and noise STRFs in terms of itBW for NRC but not for the GLM (*p<0.05 and *p>0.4*, respectively). Error bars represent SEs.

In Section 3.3 a nonparametric comparison between song and ml noise STRFs derived under a GLM or NRC showed that GLM STRFs are more similar across these two stimulus classes than are NRC STRFs. Measures of excitatory (Figure 6) and inhibitory (Figure 7) tuning taken from STRFs showed significant differences between NRC and GLM STRFs. Best excitatory and inhibitory frequency (eBF and iBF, respectively) did not differ between NRC and GLM STRFs (Figures 6A and 7A, respectively). Excitatory spectral bandwidths (eBW) were significantly different between NRC and GLM STRFs for song and noise; song NRC eBWs were larger than GLM eBWs (Figure 6B). The mean eBW for song STRFs was 1312±100 Hz for NRC and 917±41 Hz for GLM, and 703±31 and 798±35 for noise STRFs. For both song and ml noise, the difference in eBW between NRC and GLM STRFs was highly significant (*p<10^−3^*, two-sample KS test). Inhibitory spectral bandwidths (iBW) were significantly different between NRC and GLM STRFs for noise but not for song; noise GLM iBWs were larger than NRC iBWs (Figure 7B, *p<10^−3^*, two-sample KS test). Mean iBW for song STRF was 786±80 Hz for NRC and 808±35 Hz for GLM, and 603±42 and 812±31 Hz for noise. Finally, both excitatory and inhibitory temporal bandwidths (etBW and itBW, respectively) also differed between NRC and GLM STRFs (Figures 6C and 7C, respectively). For song STRFs, the mean etBW (itBW) was 9.4±0.5 (10±0.5) ms for NRC and 5.6±0.16 (5.3±1.2) ms for GLM. For ml noise STRFs, mean etBWs (itBWs) were 7.9±0.17 (8.3±0.3) ms and 5.1±0.12 (5.1±0.2) ms, respectively. For both song and ml noise, the difference in etBW and itBW between NRC and GLM STRFs was highly significant (*p<10^−3^*, two-sample KS test).

When comparing song and noise STRFs within a neuron, we found no significant differences in eBF or iBF for the NRC or GLM (Figures 6D and 7D, respectively). The differences in eBW and iBW between song and noise STRFs were significantly larger for NRC than for GLM (Figure 6E, *p<10^−3^*, and Figure 7E, *p<0.05*, two-sample KS test). Finally, we found no significant differences in etBW or itBW between noise and song GLM STRFs (Figure 6F, *p>0.1* and Figure 7F, *p>0.1*), but differences were significant for NRC STRFs (Figures 6F and 7F, *p<10^−3^*).

In summary, for the population of neurons studied here, GLM and NRC STRFs estimated from the same song and ml noise data show substantial differences. Further, ml noise and song STRFs differed significantly in their spectral and temporal properties, but differences were larger for NRC STRFs than for GLM STRFs.

### 3.5 Effects of estimation algorithm-induced biases on STRFs

As mentioned earlier, for a linear neuron, reverse correlation (RC) methods are guaranteed to produce an unbiased estimate of a neuron's true underlying STRF regardless of the stimulus statistics [36]. For a linear-nonlinear (LN) neuron, RC is guaranteed to produce an unbiased estimate of a neuron's true underlying filter only if the distribution of the stimuli used for estimation is elliptically symmetric [6]. However, in the presence of stimuli with higher-order correlations, such as zebra finch songs, RC can introduce biases in the estimate of the STRF. Something similar occurs with the GLM: if the underlying neuron behaves like a GLM, then a GLM will produce an asymptotically unbiased estimate of the STRF of the cell for any stimulus ensemble. However, any deviation from the GLM framework can introduce biases in the estimates [12].

In addition, the highly correlated structure of zebra finch songs presents additional numerical problems for STRF estimation, causing noise in the resulting STRF to be strongly amplified (see Methods for further details). Thus, some form of regularization is applied to the estimation method to obtain accurate STRFs [3], [8]–[10]. In the presence of limited or noisy data (a common scenario in neurophysiological experiments), regularization introduces a prior that constrains the STRF estimate in a way that is independent of the underlying tuning properties of the neuron, but can introduce additional biases in the STRF. Because of these types of effects, in some cases, STRFs can reflect statistical properties of the stimuli used for estimation or biases introduced by the estimation algorithm (e.g. the particular prior) rather than actual tuning properties of the underlying neuron [2], [4].

We asked whether and how much of the tuning differences we observe between song and ml noise STRFs (see Figs. 5, 6 and 7) can be explained in terms of biases introduced by the estimation algorithm. To address this, we used Kn and Ks (see Section 3.3) as LNP-type generative models to synthesize responses to both stimulus classes and re-estimate the STRFs.

Briefly, for NRC, we generated synthetic responses to song or ml noise with the following model: r_s_ = k*x_s_ +b or r_n_ = k*x_n_ +b, respectively. Similarly, for the GLM, we generated responses to both stimulus classes using Eq. 1. Here *K* and *b* (and *h*, for the case of the GLM) were either derived from recorded responses to song (Ks, b_s_) or ml noise (Kn, b_n_). Thus, we are left with two types of synthetic responses to song (r_ss_ and r_ns_), and two types of synthetic responses to noise (r_sn_ and r_nn_), which correspond to using Ks or Kn in the generative model. These four sets of responses were then used to compute two second-generation ml noise STRFs (Knn and Kns) and two second-generation song STRFs (Ksn and Kss) derived from synthetic responses to ml noise or songs, respectively. The differences between these new STRFs and the original STRFs were then quantified. Our rationale was that, if the estimation algorithms were free of biases, we should recover Kn and Ks with some added noise, regardless of the stimulus class used to re-estimate the STRFs. In particular, Knn and Kns should show small differences when compared to Kn, and Ksn and Kss should show small differences when compared to Ks.

**Figure 8:**
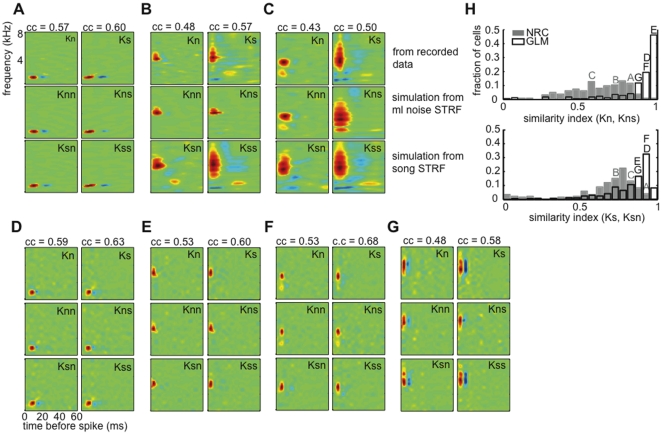
Effects of algorithm-induced biases on STRFs. (**A**)–(**C**) Top row: NRC STRFs (Kn and Ks) for the same three units shown in Figures 5A–C (c.f. Figure 5A–C, bottom row). Middle row: NRC STRFs estimated from simulated responses to ml noise (Knn) and song (Kns) when Kn is used as an LNP-type generative model for the neuron. Bottom row: NRC STRFs estimated from simulated responses to ml noise (Ksn) and song (Kss) when Ks is used as an LNP-type generative model for the neuron. (**A**) An example cell for which NRC STRFs estimated from recorded responses to ml noise (Kn) and song (Ks) and STRFs estimated from simulated responses to ml noise and song stimuli (Knn, Kns, Ksn, and Kss) do not differ. (**B**) An example cell for which Kn and Ks differ significantly. These differences cannot be explained by biases introduced by the estimation algorithm. (**C**) As in (**B**), but in this case the differences between Ks and Kn can be explained by biases introduced by the estimation algorithm, since Kns closely resembles Ks and not Kn. In addition, Ksn is more similar to Ks than Kn. (**D**)–(**F**) GLM STRFs for the same three units shown in panels A–C. (**G**) Additional example unit with broader spectral tuning and stronger inhibition. In all four examples the GLM reduces algorithm-induced biases (in all cases Kns closely resembles Kn rather than Ks and Ksn is closer to Ks than to Kn). (**H**) Distributions of similarity indices (SIs) between STRFs estimated from recorded and simulated data, for NRC (grey) and GLM (white). One indicates an exact match between STRFs and 0 indicates no correlation. Top: distribution of SIs computed between STRFs estimated from recorded responses to ml noise (Kn) and Kns (STRFs estimated from simulated responses to song using Kn as a generative model for the neuron). Bottom: distributions of SIs computed between STRFs estimated from recorded responses to song (Ks) and Ksn (STRFs estimated from simulated responses to ml noise stimuli using Ks as a generative model for the neuron). In both cases, the SI distributions for the GLM appear shifted to the right (and are centered closer to 1) when compared to the same distributions for NRC. In both cases, GLM and NRC distributions differ significantly (p<0.001, two-sample KS test). Letters indicate example STRFs.

Figure 8A–C shows the original NRC STRFs derived from recorded data and the re-estimated STRFs for the same three cells shown in Figure 5. We found that, in some cases, the underlying noise and song STRFs are recovered by the simulations (Figure 8A, compare Kn with Kns and Knn, and Ks with Ksn and Kss). However, we also found cases for which the simulated noise and song STRFs differ significantly from the ones derived from recorded responses (Figure 8B–C). In the example shown in Figure 8B, biases in the estimation algorithm are not sufficient to explain the original differences between Kn and Ks, indicating the presence of actual nonlinearities in the responses that result in stimulus-dependent tuning. In contrast, for the example shown in Figure 8C, differences between Kn and Ks can be explained by biases introduced by the estimation algorithm (that is, Kns is significantly more similar to Ks than to Kn, even though the responses used to compute Kns were originally generated from Kn).

We next repeated the same type of simulations for the GLM. Figure 8D–F shows the results of these simulations for the same three cells in Figures 8A–C, and 5. Figure 8D shows an additional example cell with broader spectral tuning and stronger inhibition. For all four examples, the GLM recovers STRFs from the simulations that are highly similar to the true underlying STRFs. Thus in these examples, it is visually clear that the GLM reduces algorithm-induced biases in the STRFs.

To quantify the amount of biases introduced in the STRFs by the GLM and NRC at the population level, we measured the similarity index (SI) between STRFs derived from recorded and simulated data. In particular, we measured the similarity between Kn and Kns (STRF estimated from synthetic responses to song when the true underlying filter in the LNP-model was Kn), and between Ks and Ksn (STRF estimated from synthetic responses to ml noise when the true underlying filter in the LNP-model was Ks). Figure 8H shows these distributions for the GLM (white) and NRC (grey) for our set of 169 cells. If the estimation algorithms introduced no (or little) bias in the STRF estimates, then the SI distribution should be, up to some variability, a narrow distribution located close to 1. For NRC, we observe a broad distribution with median  = 0.64 when we measure SIs between Kn and Kns, and with median  = 0.73 when we measure SIs between Ks and Ksn. In contrast, SI distributions for the GLM are narrower and centered closer to 1 (median  = 0.94 and 0.87, respectively), and differ significantly from NRC distributions (p<0.001, KS test, in both cases).

These analyses show that, in some cases, differences in tuning between STRFs derived from responses to song and ml noise stimuli can be explained in terms of biases introduced by the estimation algorithm, rather than actual tuning nonlinearities [2]. However, these effects are larger when NRC instead of the GLM is used.

### 3.6 Effect of the regularization prior on STRFs and predictive power

In Section 3.2 we showed that the GLM has a higher predictive power than NRC, both within and across stimulus classes (Figure 4). In addition, and in accordance with a higher across-class prediction power, we found that the GLM produces STRFs that are more similar across stimulus classes than does NRC (Figures 5, 6 and 7). We also found that, in some cases, the differences between song and noise NRC STRFs as well as the differences between NRC and GLM STRFs can be explained in terms of biases introduced in the STRFs by NRC (See Figure 8). In this Section, we addressed a related question: what component of the GLM is responsible for reducing algorithm-induced biases in the STRFs and at the same time increasing the predictive power of the model?

The GLM and NRC methods differ in three ways that result from the different assumptions about neural responses made by each of the two methods. First, the two methods optimize different objective functions; the GLM assumes point-process responses with an exponential nonlinearity while NRC assumes Gaussian noise and uses a simpler linear model. Second, the GLM and NRC use different regularization methods. Our method imposes a sparse prior on the STRF while NRC uses a lowpass Gaussian prior. Third, the GLM includes a spike history term that takes into account the recent firing probability of the neuron, while NRC does not. In principle, each of these factors may contribute to the better predictions and less-biased STRFs produced by the GLM. In order to study the effect of each component of the GL model, we removed each of these factors from the GLM framework.

**Figure 9:**
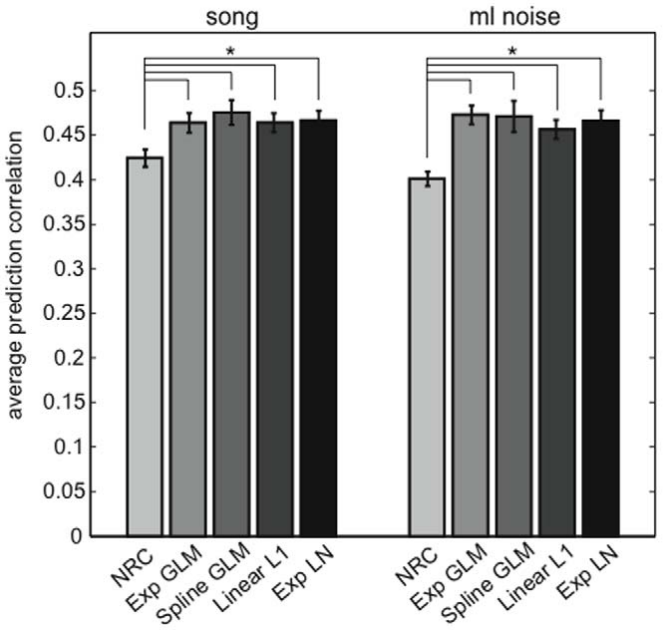
Predictive power of the different models tested. For both predictions of song and ml noise responses, all the models that use a sparse prior or L1 regularizer (i.e., Exp GLM, Exp LN, spline GLM, and Linear L1) have an average prediction correlation that is significantly higher than the average prediction correlation for NRC, which uses a smoothing prior to regularize the STRFs. We found no significant difference in predictive power across the models that employ a sparse prior. Error bars show SEs.

We first tested the hypothesis that the differences observed between NRC and GLM STRFs, and the higher predictive power of the GLM, are due to the fact that our method optimizes a different objective function than NRC. In particular, the nonlinearity employed by the GLM might be the important difference between the models. To test this, we re-fitted the GLM with a sub-exponential nonlinearity that was closer to the actual response nonlinearity in the data (see Methods). We found that this led to a slight (but statistically not significant) improvement in the predictive power and, importantly, no change in the shape of the STRFs. This weak dependence of the STRFs on the specific nonlinearity led us to ask whether it was possible to increase the predictive power of the model by fitting a cubic spline nonlinearity for each cell once the model parameters were already known (we refer to this model as spline GLM, see Methods for further details). This flexible nonlinearity conferred only a slight (but not significant) increase in predictive power for songs but not for ml noise responses when compared to the exponential GLM (see Figure 9).

Another difference between the two models is the extra term in the GLM that accounts for the neuron's spiking history. The post-spike filter could contribute to changes in the prediction accuracy of the model and in the shape of the STRF. To account for the effects of the post-spike filter on predicted responses, we re-fitted the GLM without this term, referred to here as exponential LN. We found no differences between GLM and LNP STRFs. We did, however, find that the prediction power of the GLM was slightly (but not significantly) better than the prediction power of the LN model when trying to predict responses to noise (Figure 9). It is likely that the small contribution of the spiking history to response prediction is due to the relatively low firing rate of the neurons in our sample. Spiking history may contribute to an accurate description of the detailed structure of response spike trains in higher firing rate neurons, as has been shown in the retina [15].

Finally, to test the effect of the regularization prior on predictive power and STRF shape, we fitted the same linear model used by NRC (Eq. 5), but with an L1 regularizer (Eq. 4) instead of the lowpass Gaussian prior imposed by NRC (we refer to this model as linear L1). We found that the linear L1 model has significantly higher predictive power than does NRC (see Figure 9). We found no significant difference in predictive power between the linear L1 model and the nonlinear models, with or without the history term (i.e., Exp GLM, spline GLM and Exp LN). Importantly, we found no significant differences in STRF shapes. These comparisons between STRFs and predictions generated by different models employing an L1 regularizer and NRC indicate that the differences between NRC and GLM are mostly due to the fact that the two estimation algorithms assume different priors about the STRF.

## Discussion

We used a generalized linear model (GLM) with a sparse prior to characterize the stimulus-response relationships of single auditory midbrain neurons, and compared the performance of our model to that of normalized reverse correlation (NRC) for predicting the responses to novel sounds. We found that a GLM can be successfully used to predict single-trial responses to synthetic and natural stimuli, and that, for the population of 169 cells used in this study, the GLM had a better predictive power than NRC. The performance of the GLM was better than NRC both within and across stimulus classes. The good performance of the GLM across stimulus classes suggests that our method generalizes better to changes in stimulus statistics. Differences between STRFs computed from responses to different stimulus classes (e.g. song and noise STRFs) were significantly smaller than those observed when STRFs were computed with NRC. Differences in the STRFs computed with the GLM and NRC methods were largely due to differences in the estimates of excitatory spectral bandwidths and temporal bandwidths. Below, we discuss the computational differences between the GLM and NRC that lead to differences in predictive power and STRF shapes, and compare the GLM method to other proposed methods for characterizing stimulus-response relationships in auditory neurons.

### Computational differences between the GLM with a sparse prior and NRC

As discussed before (see Section 3.6), the sparse GLM and NRC contain three fundamental differences: the two methods optimize different objective functions, use different regularization methods, and the GLM takes into account the recent firing probability of the neuron, while NRC does not. For the neurons studied here, we found that the differences in STRFs produced by both methods and the higher predictive power of the GLM are largely due to the different priors used by the two methods. Even though the GLM takes into account the cell's spiking history and uses a different nonlinearity than NRC, we found that the contribution of the spike history term as well as more complex nonlinearities led to little or no increase in the model's predictive power (see Methods and Section 3.6), and no noticeable change in STRF shape.

NRC estimates the STRF only in the stimulus subspace that contains most of the variance of the stimulus to reduce noise in the estimates and avoid overfitting [3]. With increasing levels of noise, and depending on the specific spectrotemporal characteristics of the stimulus, NRC produces STRFs that are biased towards being smooth (see Methods). This is particularly the case for songs and other natural stimuli for which the majority of the power tends to be concentrated at low spectrotemporal frequencies [40]. In this case, the spectral and temporal features at high frequencies tend to be excluded from the STRFs estimated using NRC, resulting in STRFs that substantially overestimate the contribution of low-frequency components to neural filtering [1], [4]. In contrast, the sparse GLM imposes a sparse prior on the STRFs. In this case, the amount of regularization applied to the STRF depends on the overall level of noise in the data, and in the case of a low signal-to noise ratio, GLM STRFs will be overly sparse (see Methods). Even though both NRC and GLM methods introduce biases in the STRFs, the GLM leads to better predictions and model stability across stimulus classes. However, even though the GLM leads to increased predictive power (Section 3.2), the increase we observe for our model with respect to NRC is relatively modest, if one considers the substantial differences in tuning between NRC and GLM STRFs (Section 3.5). This may seem a surprising result, since the differences in fine spectral and temporal structure of the GLM and NRC STRF do not seem to affect differences in predictive power accordingly. Nevertheless, it is important to consider that zebra finch songs, like many other natural sounds, contain relatively broadband components and change relatively slowly in time. Thus, the songs we use in this study contain relatively little power at high spectral modulation rates (i.e., modulation of stimulus power across frequency channels) and little power at high temporal modulation rates (i.e., modulation of stimulus power over time). This is why these high rates and high scales, which correspond to the stimulus domain typically excluded by NRC, and are responsible for the differences between NRC and GLM STRFs, only contribute a modest amount to prediction accuracy, as songs contain little power in that space.

### Comparison of the GLM to other methods

An alternative approach for estimating a sparse _STRF_ is boosting [41], [42]. Boosting is an estimation technique that uses coordinate ascent to minimize the number of no nzero parameters, effectively imposing a sparse prior on the STRF. [4] applied boosting on the same objective function as NRC (i.e., a linear model), to derive STRFs for primary auditory cortex neurons. Their results showed, in agreement with our findings, that boosting STRFs lead to better prediction power and show narrower spectral and temporal bandwidths than do NRC STRFs. The differences in predictive power between NRC and GLM reported here are slightly larger than the differences reported in [4] for NRC and boosting STRFs. This is presumably due to the different nonlinearities employed by the two methods. Finally, boosting can also be applied to estimate GLMs with sparse priors [42].

Several other algorithms have been developed for STRF estimation in the visual and auditory systems [8], [11], [43]. Maximally informative dimensions (MID) [11] is an information-theoretic method that finds relevant directions (a set of 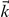 vectors, 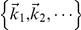) in the stimulus space. In its one dimensional version (1d-MID), this method searches for the spectrotemporal filter or STRF 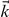, whose output, 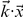, carries the most mutual information about the measured neural response *r(t)*. Once the filter is known, the nonlinearity of the LNP model is computed from the recorded data. With the GLM method, we first find the filter 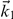 for a fixed nonlinearity (e.g. an exponential function) by maximizing the corresponding likelihood, and then use the filter to fit the output nonlinearity to the recorded data (see Methods). It has been shown that in a number of problems, including estimation of GLMs, maximizing information is equivalent to performing likelihood maximization [44]. Thus, if in the GLM method, we iterate between estimating the STRF 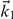 for a fixed nonlinearity and fitting the nonlinearity of the model to the recorded data, the 1d-MID and GLM methods are equivalent (except that the MID method as usually employed, does not contain any spike history terms). Here, however, we have shown that for our data set, the estimated filter is only weakly dependent on the specific form of the nonlinearity (see Methods and Section 3.6), which makes the iteration procedure in the GLM unnecessary.

Another useful method for STRF estimation is evidence optimization, introduced by [8]. This method uses a Bayesian approach to include both sparse and smooth “optimized priors” on the STRFs. These prior distributions are optimized with reference to the data, and thus they are no longer priors in the strict sense and instead become part of a hierarchical probabilistic model. The authors show that, by learning hyperparameters that control the smoothness and sparsity of the STRF in a linear model, it is possible to improve the predicting power of a model that considers only sparseness or smoothness of the estimates.

Finally, a promising future research direction is known in the statistics literature as Bayesian LASSO [45]. This method is potentially advantageous because it provides Bayesian error bars for the estimates, and is based on integrating over the posterior distribution instead of maximizing it and has some advantages in terms of how much sparsity can be enforced. This method has been previously applied [45], [46] to L1-linear regression problems but this can be easily generalized for GLMs.

### Extensions of the GLM

The same approach used by [8] to combine smoothness and sparsity priors in a linear model can be applied to a GLM. Because both smooth and sparse regularization frameworks have been shown to improve the prediction power of unregularized models [1], [3], [8], it is likely that combining features of both methods can further improve the quality of the estimates. For instance, using a prior that combines smoothness and sparsity would allow recovering smooth STRFs, while suppressing the apparent background estimation noise at high spectrotemporal frequencies.

It has been shown that the prediction performance of an LN model can be increased by using a nonlinear transformation of the stimulus (e.g., a transformation may capture nonlinearities at earlier stages of processing) that precedes the linear filtering stage [25], [47], [48]. [25] showed that a transformation of the sound stimulus using a biologically inspired model of the first stages of auditory processing [49] prior to STRF estimation with NRC led to better predictions. This model incorporates the approximate logarithmic spacing of filter center frequencies (log at high frequencies and more linear at low frequencies) in the auditory nerve and an adaptive gain control mechanism, which was important for improving the predictive power of the model. In a different approach, [47] used a learned nonlinear transform on the stimulus that converts the initial numerical representation of a stimulus value to a new representation that provides optimal input to the subsequent model. The authors apply this technique to fit an LN model to data from rodent barrel cortex, and showed that the model predicts responses to novel data accurately. Both of these two approaches can be easily applied when fitting a GLM to auditory data.

Two applications of the GLM setting are fast optimal stimulus decoding [50], and optimal stimulus design [51]. Stimulus reconstruction methods provide an important tool for understanding how sensory information is represented in neural activity. For high-dimensional stimuli such as sound spectrograms, an encoding model that suitably describes how stimuli are transformed into the spike trains of a neuron constitutes a key component for developing efficient decoding methods [52], [53]. Adaptive experimental designs, on the other hand, are particularly valuable in domains where the data are expensive or limited. This is particularly the case in STRF estimation, which requires the exploration of high-dimensional stimulus spaces, and where the inability to collect enough data has important consequences on the estimates. The GLM method described here permits the development of efficient algorithms for optimally adapting the experimental design, allowing more efficient data collection [51].
